# Estimation of Concentration and Bonding Environment of Water Dissolved in Common Solvents Using Near Infrared Absorptivity

**DOI:** 10.6028/jres.104.012

**Published:** 1999-04-01

**Authors:** Brian Dickens, Sabine H. Dickens

**Affiliations:** National Institute of Standards and Technology, Gaithersburg, MD 20899-0001; American Dental Association Health Foundation, Paffenbarger Research Center, National Institute of Standards and Technology, Gaithersburg, MD 20899-0001

**Keywords:** analysis, determination of water in solvents, hydrogen bonding distribution, near infrared spectroscopy

## Abstract

Integrated near infrared (NIR) absorbance has been used to determine the absorptivity of the *υ*_2_ + *υ*_3_ combination band of the asymmetric stretch (*υ*2) and the bending vibration (*υ*3) for water in several organic solvents. Absorptivity measured in this way is essentially constant across the absorption envelope and is found to be 336 L mol^−1^ cm^−1^ with a standard deviation of 4 L mol^−1^ cm^−1^ as estimated from a least squares fit of a straight line to data from water concentrations between 0.01 mol/L and 0.06 mol/L. Absorptivity measured from the peak maximum of the *υ*2 + *υ*3 combination band of water varies with the type of hydrogen bonding of the water molecule because the shape of the NIR absorption envelope changes with the hydrogen bonding.

Because the integrated NIR absorptivity of the *υ*_2_ + *υ*_3_ combination band of water is essentially constant across the absorption envelope, the NIR absorption envelope reflects the distribution of hydrogen bonding of the water. The shape and location of the absorption envelope appear to be governed mostly by the number of hydrogen bonds from the water molecules to easily polarized atoms. Water that is a donor in hydrogen bonds to atoms which are not easily polarized (such as the oxygen of a typical carbonyl group) absorbs near 5240 cm^−1^ to 5260 cm^−1^. Water that donates one hydrogen bond to an easily polarized atom (such as a water molecule oxygen) absorbs near 5130 cm^−1^ to 5175 cm^−1^, and water that donates two hydrogen bonds to easily polarized atoms is estimated to absorb near 5000 cm^−1^ to 5020 cm^−1^. Water donating two hydrogen bonds to other water molecules may be said to be in a water-like environment. In no case does a small amount of water absorbed in a host material appear to have a water-like environment.

## 1. Introduction

Absorbed water preferentially occupies locations where it can form hydrogen bonds. The vibrations of bonds involving hydrogen are unusual in having harmonics and combination bands with appreciable absorbance in the near infrared (NIR) region of the spectrum. Because hydrogen is a light atom, its bonds have high vibrational frequencies (roughly 2800 cm^−1^ to 3600 cm^−1^ for the fundamentals of the stretching vibrations). The fundamental stretching vibrations of most other bonds lie below 2000 cm^−1^. The low harmonics of bonds containing hydrogen lie in the NIR. Only higher harmonics of the other vibrations have such high frequencies. Higher harmonics are less absorbing than lower harmonics. The NIR region is particularly useful in studying the IR absorptions of bonds containing hydrogen [1]. The strongest NIR absorption for water, the *υ*_2_ + *υ*_3_ combination band of the asymmetric stretch (*υ*_2_) and the bending vibration (*υ*_3_), is at about 5200 cm^−1^. The first overtone of the symmetric stretch (*υ*_1_), at about 7140 cm^−1^, is significantly weaker than the *υ*_2_ + *υ*_3_ combination band but can often be discerned.

The NIR is weakly absorbed in general and then mostly by hydrogen-containing bonds. This greatly simplifies the NIR absorption spectrum over that of the middle IR, where the absorptions are in general due to fundamental vibrations rather than overtones and many more bonds than those involving hydrogen absorb. Because of its low absorption, the NIR can be used to examine specimens much thicker (from about 25 mm up to at least 1 cm) than the 1 mm to 10 mm thickness commonly required for the middle IR region. In particular, use of the NIR allows examination of water in free-standing solid materials such as thick films and disks.

The strength of hydrogen bonding of the water can be inferred from the frequency of the O–H absorbance. When hydrogen bonding occurs to produce a configuration such as O_D_–H⋯O_A_, where O_D_ is the donor and O_A_ the acceptor in the hydrogen bond, the covalent O_D_–H bond distance increases and the O_D_⋯O_A_ inter-oxygen distance decreases to become less than the sum of the van der Waals radii for oxygen [2]. The polarity of the O_D_–H bond increases over that of a similar bond in an isolated molecule. These perturbations are reflected in the vibrational frequency of the O–H bond and are observable in the NIR. In the most extreme case, that of liquid water, the complete absorption envelope for the *υ*_2_ + *υ*_3_ absorption is from about 4600 cm^−1^ to 5400 cm^−1^, as shown below. Most of this region, and certainly the part covering the *υ*_2_ + *υ*_3_ absorption from low quantities of water absorbed in a host, is a convenient window in many spectra.

The amount of moisture absorbed by materials is important in many areas of technology because of the influence of moisture on mechanical properties, electrical properties, adhesion, corrosion, hydrolysis reactions, etc. Measurements of absorbance in the NIR can be useful not only for deducing the concentration of water but also for providing information about the nature of hydrogen bonding of the water to its surroundings. This paper explores the use of NIR spectroscopy to assess the concentration and environment of water dissolved in several common solvents.

## 2. Experimental

The NIR absorptivity-water concentration relationship was examined using a series of solvents as host matrices for water. Solvents were used as hosts because known amounts of water could easily be added volumetrically.

The NIR measurements were carried out with a Nicolet Magna-IR 550 spectrometer (Nicolet Inc., Madison, WI)^1^ equipped with a KBr beam-splitter and an MCT-A^2^ detector cooled with liquid nitrogen. The spectrometer was flushed continuously with nitrogen gas newly boiled from a liquid nitrogen source. Spectra were measured using 32 scans at 2 cm^−1^ resolution.

The solvents were toluene, carbonyl-group-containing solvents such as a ketone with enolic forms (acetone), a ketone with fewer enolic forms (methyl ethyl ketone, MEK), an ester (ethyl acetate), an acid (acetic acid), and a solvent containing ketone and amine groups (*N*-methyl pyrrolidinone, NMP). All but toluene represent hydrophilic environments.

Several trials were necessary to establish workable techniques. Initially, the solvents were dried over calcium sulfate [3]. However, water could not be discerned in dried toluene decanted from calcium sulfate until 3 μL of water per 50 mL toluene had been added, so the solvent was distilled to remove traces of the drying agent. A white solid, presumably calcium sulfate, remained after each distillation. “Dry” acetone, MEK, ethyl acetate, and acetic acid were also produced by distilling the solvents over anhydrous calcium sulfate. NMP was used as newly purchased because it has such a high boiling point that it can not be distilled easily. Decomposition occurs if the pressure is not reduced sufficiently.

Except for toluene, water-solvent mixtures were first made by taking dry solvent and incrementally adding water to the solution after taking the NIR spectrum. Satisfactory results were obtained for water in toluene when aliquots of water were added to dried distilled toluene in 50 mL graduated flasks which were allowed to stand overnight at room temperature before the NIR spectra were taken.

To investigate the behavior of water at the lowest concentrations, a more precise technique for measuring NIR absorbance of low amounts of water in hydrophilic solvents was developed for ethyl acetate and acetone. The solvents were dried with heat-activated 3A molecular sieves for at least 2 days, after which the molecular sieve was filtered off under vacuum with a 0.2 mm fluorocarbon filter. To make those solutions containing the smallest amounts of water, a scheme of volumetrically diluting a water-solvent solution was used. Attempts to remove all the sieve material by filtering the solvent appeared to be unsuccessful because inconsistent results were obtained when NIR spectra were taken of solutions containing only a few microliters of water per 10 mL of solvent.

A scheme was then devised wherein the solvent was dried over 3A molecular sieves, then decanted under argon into a flask attached to a vacuum line. Argon was used because it is much heavier than air and exerts a blanketing effect. The pressure in the vacuum line was reduced until the solvent distilled at room temperature into a receiving flask cooled with ice water. After distillation, about 10 mL of solvent (sufficient to fill the cell at the longest path length) was transferred under argon into each of a series of preweighed 20 mL vials and the required microliter quantities of water or water-containing solvent were added through a septum with a hypodermic syringe. Solutions with less than 10 mL of water in 10 mL of solvent were obtained by adding aliquots of small amounts of water in dry solvent to the dry solvent in the vials.

Absorption of water on the vial walls would clearly affect those solutions containing low quantities of water. To minimize such perturbations, the vials were first dried at > 100 °C, cooled, and allowed to equilibrate with room air before being flushed briefly with argon. They were then capped with a septum held in place by a crimped ring and weighed. The rationale for this sequence was to allow an equilibrium film of absorbed water to form and remain on the walls of the vials but to flush out humid air. Great care was taken to ensure that head spaces above the solvent-water mixtures in the vials, the IR cell itself, and the syringes used to fill the IR cell were always flushed and filled by dried argon gas. Later NIR measurements showed that the mixtures in the vials retained their original levels of water for at least several days.

Specimens were prepared in this way for acetone and for ethyl acetate. Mostly, spectra over the range of 4000 cm^−1^ to 7500 cm^−1^ were ratioed against spectra of the dry solvent taken at the same cell path length. Cell path lengths from 7 mm to 0.2 mm were used, depending on the amount of water. In the case of acetone, water-acetone mixtures with water volume fractions ranging from 0 to 1 were studied. The NIR spectrum of acetone has an absorption at about 5100 cm^−1^, which may be from the third harmonic of the carbonyl group or from enolic forms. In order to remove mismatch between the sample spectra and reference spectra, a reference spectrum of CCl_4_ was used when measuring the spectra of acetone-water mixtures containing volume fractions of water < 10 %.^3^ Tests were carried out to ensure that the NIR beam was essentially constant from day to day over a period of several days and that the sample of CCl_4_ used had no NIR absorption in the ranges of interest. Comparison of NIR spectra suggested that reasonably good measurements of small quantities of water were obtained.

## 3. Results and Discussion

### 3.1 Toluene

**Fig. 1:**
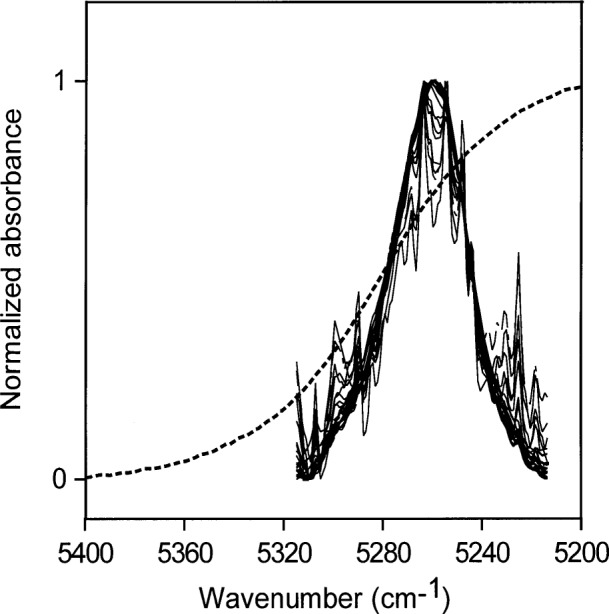
Spectrum of liquid water (dotted line) and NIR spectra of various amounts of water, up to the solubility level, dispersed in toluene and normalized to a maximum value of one. The constancy in shape of the absorbances of water in toluene shows that the water molecules have the same hydrogen-bonding environments at all these solubility levels.

**Fig. 2:**
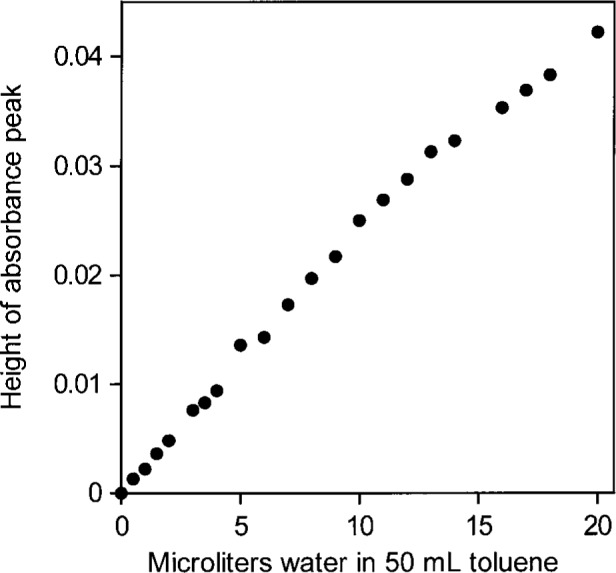
Peak heights of the NIR *υ*_2_ + *υ*_3_ combination band absorbance envelope for water in toluene versus amount of added water.

Using peak heights for water in toluene gives an NIR absorptivity of 3.08 L mol^−1^ cm^−1^ and leads to an estimate of 0.024 mol (0.44 mL) water per liter in toluene saturated with water. The NIR spectral peaks all have the same shape (Fig. 1). Therefore, it is possible and reasonable to use the peak maximum as a measure of the water absorbance, shown in Fig. 2 to be linear with water content. From the “high” value (3.08 L mol^−1^ cm^−1^) of the absorptivity and the constancy of the shape of the water NIR absorption envelope, it appears that the water molecules are isolated from one another in this environment, consistent with their low abundance. However, the apparent absorptivities in Table 1 calculated from heights of NIR peak maxima for water in various solvents show no convincing relationship between absorptivities, location of the peak maxima in terms of wavenumber and water concentration.

Because the intent was to use NIR spectra to quantify the amount of water present, even though the peak maximum-derived absorptivity varies with the degree of hydrogen bonding, checks were made to see if the absorptivity varies significantly across the NIR absorption envelope. If so, it would be necessary to devise some series of experiments to measure absorptivities in different parts of the envelope.

**Fig. 3:**
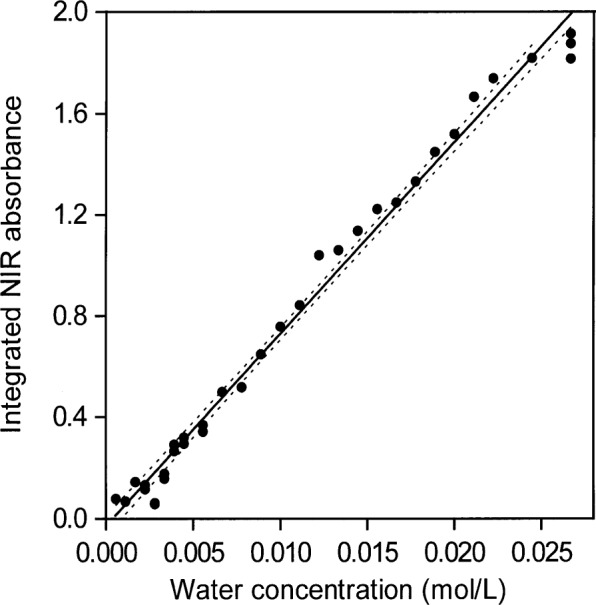
Peak areas of the NIR *υ*_2_ + *υ*_3_ combination band absorbance envelope for water in toluene versus amount of added water. The straight line regression line and the 95 % confidence intervals are shown. Areas were integrated with the NIR spectra plotted in terms of wavenumbers.

When areas under the peaks (peak areas) were used to provide absorptivities, it was found that all integrated absorbance-water concentration pairs from all solvents lie more or less on the same straight line. This shows that water absorptivity is essentially constant at all wavenumbers in the NIR absorption envelope although the shape of the envelope changes with the type of hydrogen bonding. When the shape of the absorption envelope changes considerably, the conventional technique of estimating absorptivity from the peak height is not valid. A plot of peak area versus increasing concentrations of water in toluene is shown in Fig. 3. At such low water concentrations, the peak area measurements are not as precise as the peak maxima measurements (Fig. 2) because the low signal-to-noise ratio at the edge of the absorption envelope makes estimating the baseline difficult. However, it is feasible to use both peak height and peak area measurements for toluene because the absorption envelope does not change shape with increasing water content (Fig. 1).

The finding that absorptivity is essentially constant at all wavenumbers in the NIR absorption envelope of the *υ*_2_ + *υ*_3_ combination band has the considerable benefit that the NIR absorption envelope is essentially a representation of the distribution of hydrogen bonding of water. The water must of course be present as water molecules rather than in some reacted form and there must be no other significant source of O–H bonds, such as alcohol or enolic forms of ketones, in the system. Thus, NIR spectroscopy can be used to provide simple comparisons of hydrogen-bonding distributions of water in selected environments. In the examples shown here, NIR absorbance was integrated with the abscissa of the NIR spectrum plotted in wavenumbers.

When using peak areas, the baseline must be well-known across a wide range. Noise becomes more important as the absorbance becomes small, i.e., for low water contents, especially in the wings of the absorption envelope. Also, the area-derived absorptivities are quite different in scale from the peak height absorptivities normally reported, and the two can not easily be related.

### 3.2 Acetone

**Fig. 4:**
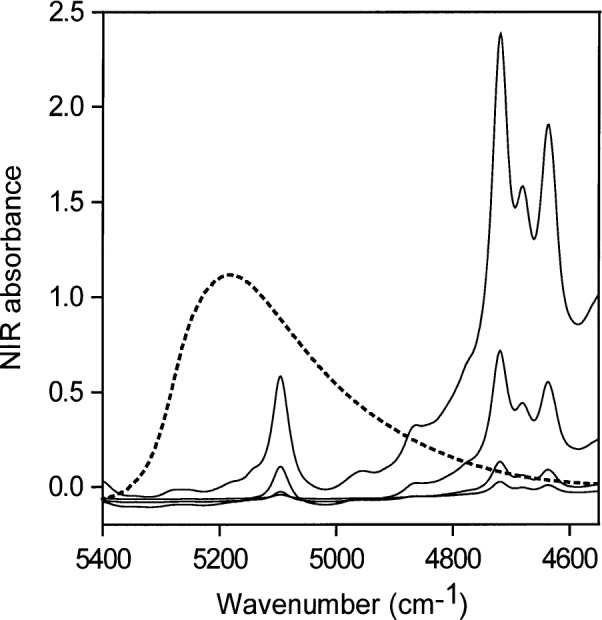
NIR spectra of pure water (dotted line) taken with a path length of 0.2 mm and of pure acetone taken with path lengths of 0.2 mm, 0.5 mm, 2 mm and 6 mm. The acetone water absorbance peak appears at about 5100 cm^−1^.

**Fig. 5:**
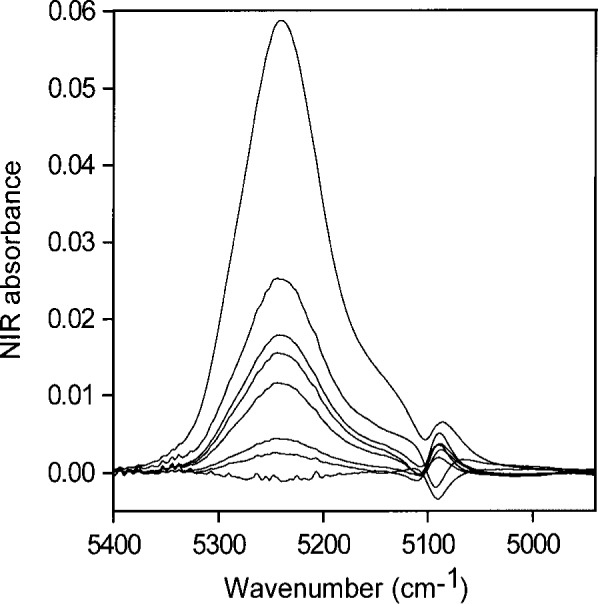
NIR spectra for the lowest concentrations of water in acetone (0.001 mol/L to 0.03 mol/L water), acetone blank subtracted.

Figure 4 contains NIR spectra of pure water taken with a path length of 0.2 mm, and of pure acetone taken with path lengths of 0.2 mm, 0.5 mm, 2 mm, and 6 mm. The spectra of water and acetone taken with 0.2 mm path length are therefore directly comparable and show that the acetone carbonyl absorption at about 5100 cm^−1^ is negligible compared with that of pure water in this region. The acetone carbonyl absorption becomes more important if acetone-water solutions with large amounts of water are ratioed against pure acetone, if the cell path lengths are not set accurately, and especially if the water concentration is very low. Mismatches of acetone spectra account for the appearance of some perturbation at 5100 cm^−1^ in the acetone-water spectra at low water concentrations (Fig. 5).

**Fig. 6:**
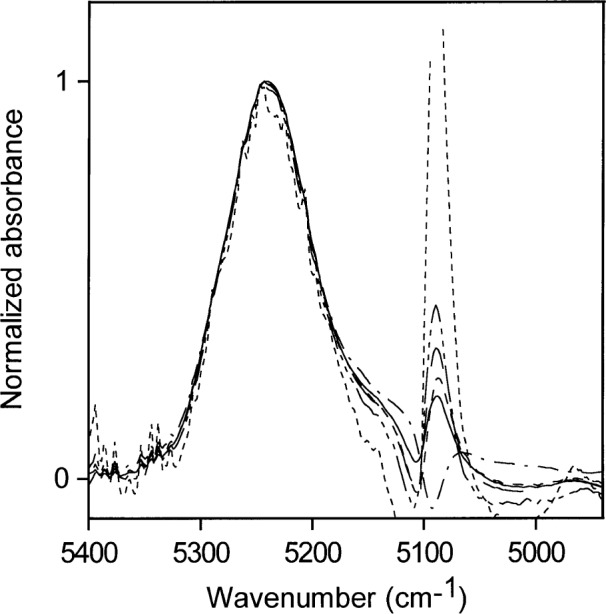
Normalized versions of the spectra in Fig. 5 for the lowest concentrations of water in acetone (0.001 mol/L to 0.03 mol/L).

The NIR spectra for the lowest concentrations of water in acetone (0.001 mol/L to 0.03 mol/L) normalized to a maximum height of 1 are shown in Fig. 6. Because all the absorptivities contributing to the envelope are the same, Fig. 6 allows comparison of the distribution of hydrogen bonding as the water concentration changes and shows that the shape of the NIR absorption envelope and hence the shape of the hydrogen bonding distribution are constant with changing water concentration at these low water concentrations.

Figure 6 also shows the difficulty of obtaining good estimates of the area of the water absorption at such low water concentrations because the acetone carbonyl peak makes a considerable contribution to the total area. For ease in data handling, areas in the current study were all estimated over the same wave number range for a given water concentration range. An individual approach to each spectrum is necessary to provide the best quantitative estimates at these low concentrations but the purpose of this study did not justify such a detailed examination.

**Fig. 7:**
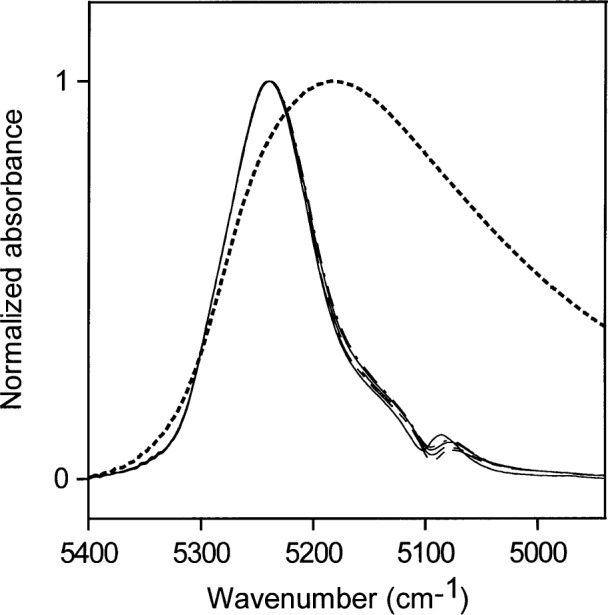
Normalized NIR spectra for medium-low concentrations of water in acetone (0.03 mol/L to 0.52 mol/L). The NIR absorbance of liquid water is shown as a dotted line.

Figure 7 shows NIR spectra for medium-high concentrations of water in acetone (0.03 mol/L to 0.52 mol/L) normalized to a maximum height of 1. The relative contributions of the acetone carbonyl absorptions are much smaller than in Figs. 5 and 6. Figure 7 shows that the spectra do not overlap exactly, which means that there are small changes in the hydrogen-bonding scheme over this concentration range. For comparison, the normalized NIR spectrum of pure water is superimposed on the normalized acetone-water spectra. The absorption envelope of water in acetone at these concentrations lies at the “nonhydrogen-bonding” end of the water hydrogen-bonding distribution. However, if the water were not hydrogen-bonded, it would not dissolve in acetone at concentrations above those found for water in toluene. Therefore, most of the water at these concentrations must be hydrogen-bonded to the carbonyl group of acetone, where the water is the donor in the hydrogen bonds, and can not be hydrogen-bonded to other water molecules, which would then be acceptors in hydrogen bonds. The carbonyl group is not easily polarized and does not affect the O–H bond very much. Therefore, the term “non-perturbed” will be used in referring to water molecules that absorb in the 5200 cm^−1^ region of the NIR.

Shifts in the NIR spectrum towards 5100 cm^−1^ reveal shifts in the hydrogen-bonding distribution, which are a consequence of water hydrogen bonding to other water molecules, whose oxygen atoms are more easily polarized than the oxygen atoms in carbonyl groups. Hydrogen bonding between water molecules will occur where there is enough water present for some of the water to compete with the host material as acceptor of hydrogen bonds. The hydrogen-bonding scheme indicated by Fig. 7 is consistent with the picture that at the highest concentration the abundance of water is about 1 water molecule for every 3.5 acetone molecules. At this water concentration, some water molecules must have other water molecules as neighbors to account for the slight increase in water molecules acting as acceptors of hydrogen bonds. As the water concentration increases, a broadening of the NIR absorbance is seen.

**Fig. 8:**
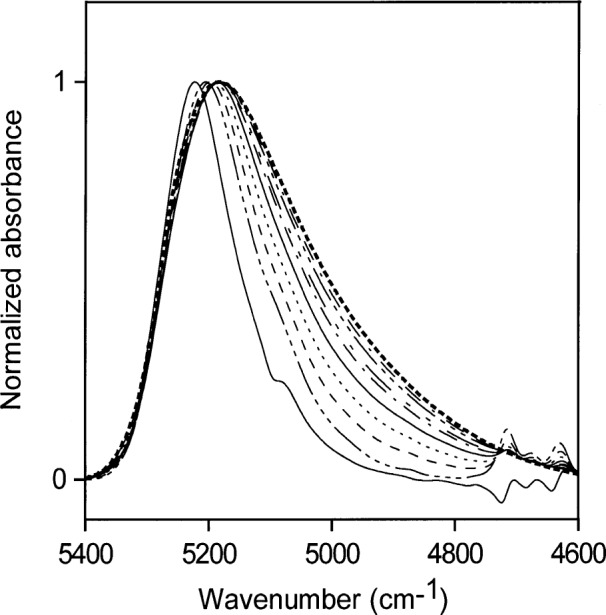
Normalized NIR spectra for acetone:water solutions with volume fractions of water from 0.1 to 1 (5.6 mol/L to 55.6 mol/L) in steps of 0.1. The contribution of acetone has been subtracted. The right-most curve (dotted line) is the NIR spectrum of liquid water.

Figure 8 shows the corresponding normalized spectra for acetone water solutions with volume fractions of water ranging from 0.1 to 1.0 (5.6 mol/L to 55.6 mol/L) in steps of 0.1. The right-most curve in Fig. 8 (dotted line) is that of pure water. The NIR absorption shifts reveal the changes in hydrogen-bonding distribution as the hydrogen-bonding scheme of the dissolved water moves toward that in liquid water. Table 2 shows the changes in molecular abundance of water and acetone molecules for the curves in Fig. 8.

### 3.3 Methyl Ethyl Ketone (MEK)

**Fig. 9:**
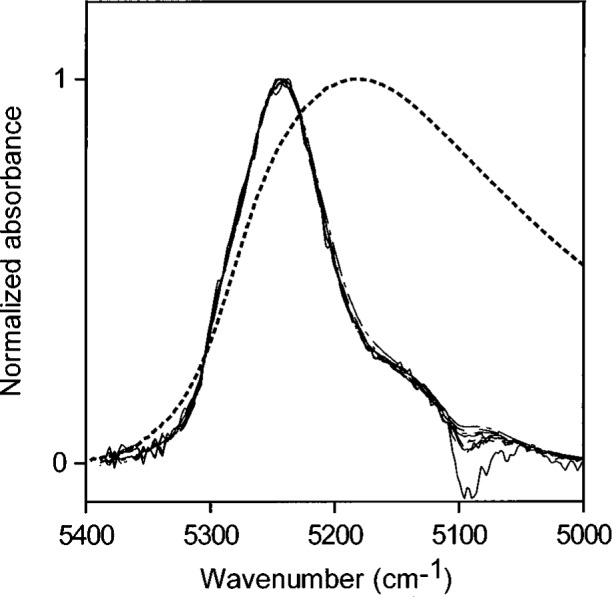
Normalized NIR spectra of liquid water and low water concentrations (0.002 mol/L to 0.33 mol/L) in methyl ethyl ketone. The dotted line is the normalized NIR spectrum of liquid water.

**Fig. 10:**
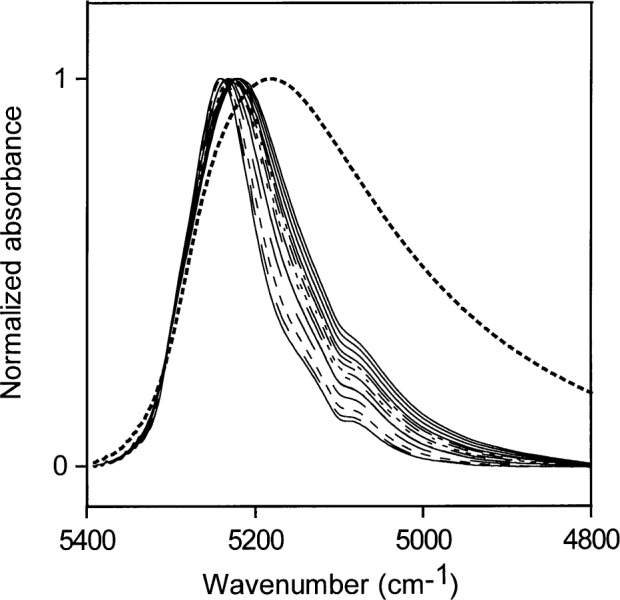
Normalized NIR spectra for higher water concentrations (0.56 mol/L to 7.78 mol/L) in methyl ethyl ketone. The dotted line shows the normalized spectrum of liquid water.

Normalized spectra for low water concentrations (0.002 mol/L to 0.33 mol/L) in MEK are shown in Fig. 9. The bonding scheme at these low water concentrations changes only slightly, but the maximum ratio of water molecules to MEK molecules for these concentrations is only 1 water molecule for 20 MEK molecules and not much inter-water hydrogen bonding is expected. Spectra from higher water concentrations (0.56 mol/L to 7.78 mol/L) are displayed in Fig. 10 (7.78 mol/L water is near the saturation point of MEK). This corresponds to a molecular ratio of about 1.3 MEK molecules for every water molecule. The hydrogen bonding of the water is preferentially to the carbonyl groups of MEK. However, the NIR spectra in Fig. 10 show that the oxygen atoms of water increasingly become acceptors in hydrogen bonding as the water concentration is increased over the range from 0.56 mol/L to 7.78 mol/L.

### 3.4 Ethyl Acetate

**Fig. 11:**
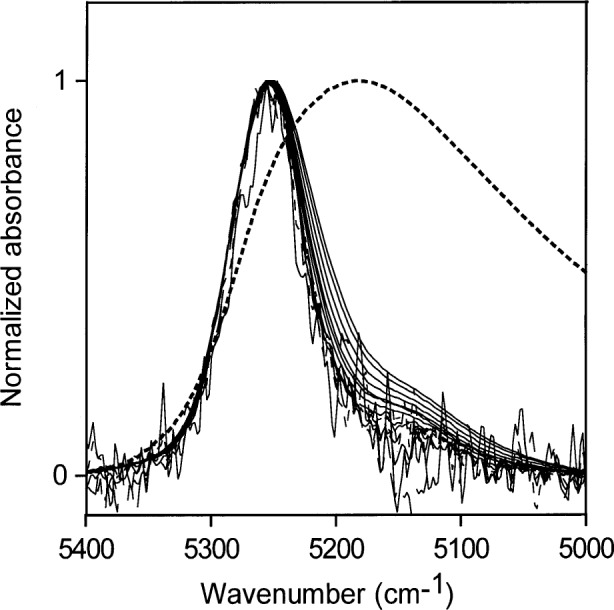
Normalized NIR spectra for ethyl acetate:water solutions up to the saturation limit. The dotted line is the NIR spectrum of liquid water.

The saturation limit of water in ethyl acetate is about 1.5 mol/L water. Normalized NIR spectra of ethyl acetate-water solutions up to this limit are shown in Fig. 11. The NIR absorption envelope of water in ethyl acetate shows that most of the water molecules are at the non-perturbed end of the hydrogen-bonding distribution, as in acetone at low water concentrations. The development of some small amount of water-water hydrogen bonding with increasing water concentrations is evident. Water in ethyl acetate must be predominantly hydrogen bonded to the ester group, probably mostly to the carbonyl part of the ester group. Ethyl acetate is less hydrophilic than acetone, and at a ratio of about 1:6 water:ethyl acetate molecules the saturation point of water in ethyl acetate is reached.

### 3.5 Acetic Acid

**Fig. 12:**
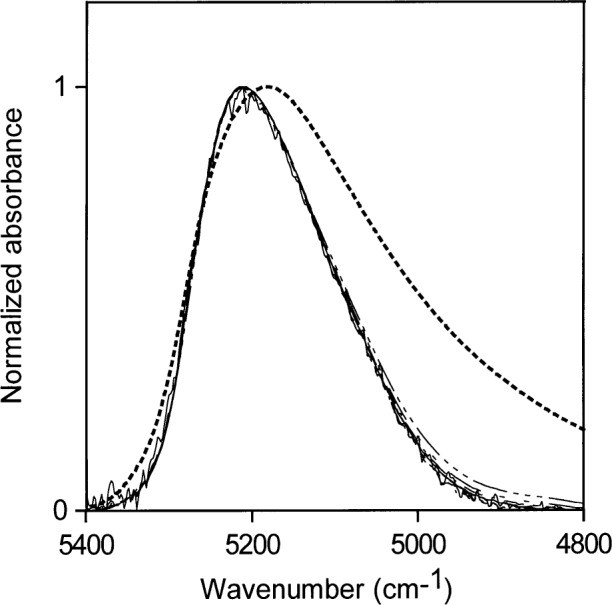
Normalized NIR spectra of up to 4 mL water in 50 mL acetic acid (water concentrations of 0.056 mol/L to 4.44 mol/L water). The normalized spectrum of liquid water is shown as a dotted line.

The normalized absorption envelope of water and the normalized NIR spectra of a series of solutions of water in acetic acid, ranging from water concentrations of 0.056 mol/L to 4.44 mol/L, are shown in Fig. 12. The hydrogen bonding of water in acetic acid does not change much over this concentration range, which corresponds to a minimum of 4 acetic acid molecules for every water molecule. The hydrogen-bonding distribution is wider than those of water at low concentrations in acetone and in ethyl acetate, but much of the water in acetic acid at these fairly low concentrations (in terms of molecular ratios) must be the donor in its hydrogen bonds because the NIR absorption envelope of water in acetic acid includes the non-perturbed end of the absorption envelope of pure water.

### 3.6 *N*-Methyl Pyrrolidinone (NMP)

**Fig. 13:**
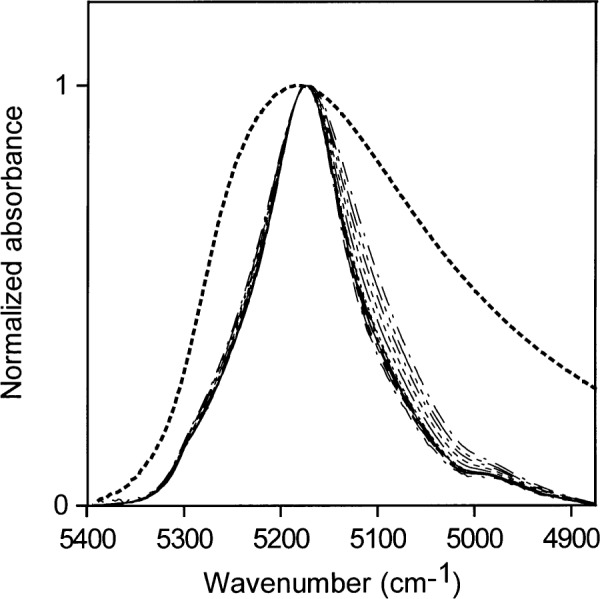
Normalized NIR spectra for liquid water (dotted line) and water concentrations of 0.11 mol/L to 3.89 mol/L in *N*-methyl pyrrolidinone.

Spectra for water concentrations of 0.11 mol/L to 3.89 mol/L in NMP are shown in Fig. 13. These spectra are the first example in this study of a water absorption at low to medium-low concentrations in an organic solvent not being at the non-perturbed end of the liquid water spectrum. The peak maximum of the NIR absorption envelope is at ≈ 5175 cm^−1^. The hydrogen bonding of the water molecule in NMP places the water molecule in the middle of the hydrogen-bonding distribution of liquid water, where the number of accepted hydrogen bonds ranges from zero to two. (Because the hydrogen bonding of water in NMP is at neither end of the liquid water range, it seems reasonable to describe it as similar to a water molecule donating one hydrogen bond to another water molecule.) However, water present in NMP in low concentration must be hydrogen-bonded to the NMP molecules. A reasonable explanation is that the water is hydrogen bonded to the nitrogen atom in NMP or, if it is bonded to the carbonyl group, that the electronic distribution in the carbonyl group in NMP is much denser than that in acetone. In either case, the electron distribution of the accepting group in NMP appears to resemble that of an oxygen atom in a water molecule.

### 3.7 All Solvents

**Fig. 14:**
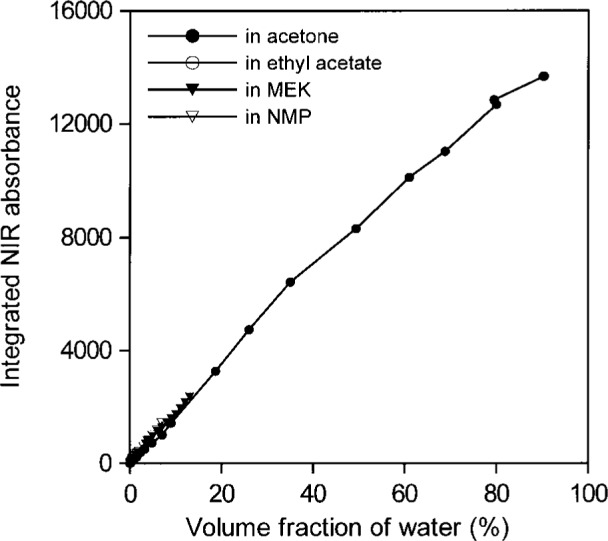
Integrated NIR water absorbance for water in acetone, ethyl acetate, methyl ethyl ketone (MEK), and *N*-methyl pyrrolidinone (NMP) as a function of water content from volume fractions of 0.00001 to 1.

**Fig. 15:**
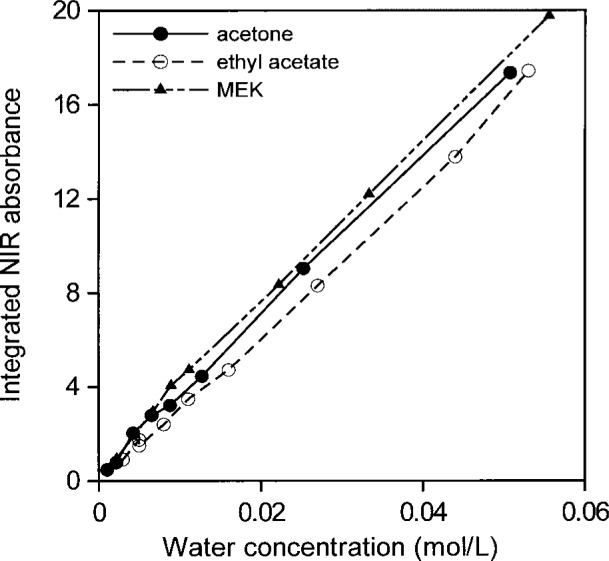
Integrated NIR water absorbance for water in acetone, ethyl acetate, and methyl ethyl ketone (MEK) as a function of water concentration from 0.001 mol/L to 0.06 mol/L (i.e., up to a volume fraction of 0.0011).

Figures 14 and 15 show that all areas of water absorption envelopes correlate well with all water contents for all solvents (*R*_2_ = 0.997). For NMP, it seems that moisture in the atmosphere affected the lowest water concentrations. In support of that view, the first measurement for NMP, at the lowest water concentration, seems to have been correct. Otherwise, differences among the curves, more evident in Fig. 15, are probably mainly due to problems in subtracting spectra and in removing baselines in the peak integration process. For that reason, the average absorptivity based on integrated NIR areas for water in solvents was estimated from water concentrations between 0.01 mol/L and 0.06 mol/L. The integrated absorptivity calculated from mixtures of water with acetone, ethyl acetate, and MEK was found from a least squares fit of a straight line to the data to be 336 L mol^−1^ cm^−1^ with a standard deviation of 4 L mol^−1^ cm^−1^.

### 3.8 Environment of Water Molecules from NIR Measurements

Water is not completely hydrogen bonded, even in almost freezing liquid, and the bonding decreases with increasing temperature [4 and 5], presumably to become somewhat like the distribution in toluene, shown together with the normalized NIR spectrum of liquid water in Fig. 1. Goldstein and Penner [5] assigned 5220 cm^−1^, 5120 cm^−1^, and 5000 cm^−1^ to the peak positions of the NIR absorbances for water molecules in liquid water involved in 0, 1, and 2 hydrogen bonds, respectively.

**Fig. 16:**
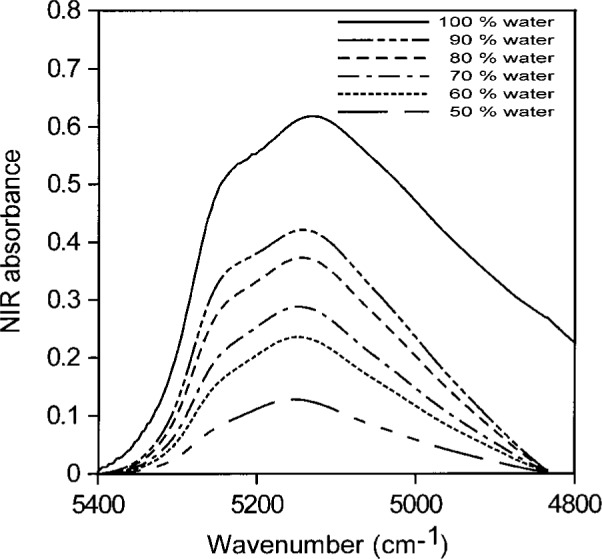
Subtracted spectra: the spectrum of 40 % volume fraction of water in acetone was subtracted from the spectra of 50 %, 60 %, 70 %, 80 %, and 90 % volume fraction of water in acetone and from the spectrum of pure water.

**Fig. 17:**
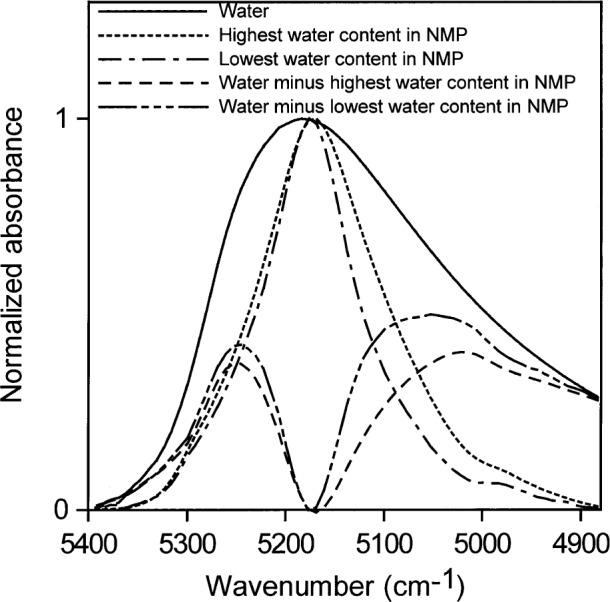
The spectral contributions of water in zero (maximum at (≈ 5250 cm^−1^) and two perturbing hydrogen bonds (maximum at (≈ 5050 cm^−1^), obtained by subtracting the spectrum of water in *N*-methyl pyrrolidinone (NMP) from that of pure water.

All of our measurements of the distributions of hydrogen bonding of water in various environments were taken at room temperature (about 23 °C). Water in toluene (accepting no hydrogen bonds) absorbs with the peak maximum at ≈ 5260 cm^−1^. Water in NMP (accepting one hydrogen bond) absorbs with the peak maximum at ≈ 5175 cm^−1^. Subtracting 40 % water in acetone from higher concentrations of water in acetone gives a shoulder at ≈ 5240 cm^−1^ and a peak at ≈ 5130 cm^−1^ (Fig. 16). A solution with a volume fraction of 40 % water in acetone contains about 2.7 water molecules per acetone molecule (see Table 2) and each water molecule on average has at least one water molecule as a neighbor. Therefore, each water molecule statistically hydrogen bonds to approximately one neighboring water molecule. Assigning absorptions at ≈ 5240 cm^−1^ to ≈ 5260 cm^−1^ to non-perturbing hydrogen bonds and ≈ 5130 cm^−1^ to ≈ 5175 cm^−1^ to 1 “perturbing” hydrogen bond is consistent with the assignments of Goldstein and Penner. A “perturbing” hydrogen bond is a hydrogen bond to an easily polarized atom such as a water oxygen. A very slight hint of a shoulder at 5000 cm^−1^ in these spectra may reflect where the peak maximum of water involved in two perturbing hydrogen bonds is to be found, i.e., two hydrogen bonds to water oxygens or their electronic equivalents. A second way of estimating these peak centers is to subtract the normalized spectrum of water in NMP from the normalized spectrum of pure water, as in Fig. 17. The resultant peak maxima are at ≈ 5247 cm^−1^ for water with no perturbing hydrogen bonds and ≈ 5040 cm^−1^ to ≈ 5055 cm^−1^ for water with two perturbing hydrogen bonds. The water absorption envelope is non-zero to at least 4800 cm^−1^.

## 4. Conclusions

The shape of the NIR absorption envelope reflects the H-bonding of the dissolved water molecules. Although the shape of the absorption band due to absorbed or dissolved water in a variety of solvents changes with the quantity of water, the integrated intensity from 5400 cm^−1^ to 4800 cm^−1^ can be correlated to concentration with an absorptivity of 336 L mol^−1^ cm^−1^ with a standard deviation of 4 L mol^−1^ cm^−1^ as estimated from a least squares fit of a straight line to data from water concentrations between 0.01 mol/L and 0.06 mol/L. From the position of the absorbance maximum, we conclude that hydrogen bonding of water to toluene, ketones, and esters perturbs the O–H frequency of the water molecule only slightly. Water in *N*-methyl pyrrolidinone (NMP) appears to make one hydrogen bond to a group in NMP which acts like a water oxygen in perturbing the frequency of the hydrogen bond. In none of the solvents examined does a small amount of water appear to have a water-like environment. Using peak area absorptivities allows water content to be rapidly and conveniently estimated in any solvent where the water peak can be discerned in the NIR spectrum. The apparent constancy of the peak-area absorptivity makes calibration at low water concentrations unnecessary because estimates of the water absorptivity can be obtained at higher water concentrations.

## Figures and Tables

**Table 1:** NIR maxima of the *υ*_2_ + *υ*_3_ water combination band; single measurement data. The standard deviations^a^ are estimated to be less than 5 % of the values

Host solvent	Concentration range of water (mol/L)	Absorptivity calculated from peak height	Position of NIR maximum (wavenumber, cm^−1^)
Toluene	0–0.024	3.1	5260
Acetone	0–22	1.2–3.1	5190–5237
Methyl ethyl ketone	0–7.2	1.4–3.3	5219–5238
Ethyl acetate	0–0.56	3.3	5255
Acetic acid	0–4.4	1.4	5149
*N*-methyl pyrrolidinone (NMP)	0–3.3	2.7–3.3	5175
(NMP)			

a All standard deviations in this paper were obtained from the measurements and are not to be taken as a complete statement of uncertainty.

**Table 2:** Calculated molecular abundance of water and acetone molecules for idealized mixtures corresponding to the curves in Fig. 8

Volume fraction of water	Molecules of acetone per water molecule
0.1	2.21
0.2	0.98
0.3	0.57
0.4	0.37
0.5	0.25
0.6	0.16
0.7	0.11
0.8	0.06
0.9	0.03
1.0	0.00

## Footnotes

1 Certain commercial equipment, instruments, or materials are identified in this paper to foster understanding. Such identification does not imply recommendation or endorsement by the National Institute of Standards and Technology, or the American Dental Association Health Foundation, nor does it imply that the materials or equipment identified are necessarily the best available for the purpose.

2 MCT = mercury cadmium telluride.

3 All quantities given in % are volume fractions.
